# Factors associated with a measles outbreak in three health districts of Cameroon in 2019: a cross-sectional study

**DOI:** 10.11604/pamj.2023.46.41.35832

**Published:** 2023-09-28

**Authors:** Esum Mathias Eyong, Andreas Ateke Njoh, Sophie Jose Molua Etutu, Hassan Ben Bachir, Shalom Tchokfe Ndoula, Yauba Saidou, Samuel Wanji

**Affiliations:** 1 Department of Microbiology and Parasitology, Faculty of Science, University of Buea, Buea Cameroon,; 2 School of Global Health and Bioethics, Euclid University, Bangui, Central African Republic,; 3 Expanded program on immunization, Ministry of Public Health, Yaoundé, Cameroon,; 4 Department of Family Health, Ministry of Public Health, Yaoundé, Cameroon,; 5 Clinton Health Access Initiative, Yaoundé, Cameroon,; 6 Institute of Global Health, University of Siena, Siena, Italy

**Keywords:** Measles, outbreak, vaccine, armed conflict, temperature, South West Region, Cameroon

## Abstract

**Introduction:**

measles is an infectious viral disease that affects susceptible individuals of all ages. It is a leading cause of death among young children globally due to suboptimal vaccination coverage. In 2019, measles outbreaks affected several parts of the world, including three health districts (HDs) of Cameroon's South West Region (SWR) experiencing armed conflict. Herein, we assessed the factors associated with the outbreak in the SWR.

**Methods:**

we conducted a comparative study from March to August 2020. Data on study participants were compared between the three HDs that experienced a measles outbreak and three other HDs of the region that reported a case of measles but did not get into an outbreak. Records on vaccination between 2015 and 2019 were reviewed.

**Results:**

information was obtained from 56 participants with known measles status, 32 from outbreak districts, and 24 from non-outbreak districts. The population in the outbreak group was more likely to have traveled from an area in a measles outbreak (OR 2, 95%CI 1.1-11.20). There was a suboptimal availability of measles vaccines in both categories of districts compared to the needs, and there was a downward trend in vaccination coverage in both groups. In addition, vaccines were more exposed to extreme temperatures in HDs with the outbreak (P<0.01) from 2015 to 2019. We found no statistically significant difference between both groups concerning the preexisting comorbidities of participants.

**Conclusion:**

there is an urgent need to improve the cold chain and intensify vaccination activities in these districts.

## Introduction

Measles is an infectious disease caused by the measles virus [1,2]. It affects susceptive individuals of all ages [1]. Unimmunized infants are particularly at risk of measles and its complications [2]. However, this disease is entirely preventable through two safe and effective measles vaccine doses [3]. Unfortunately, the global coverage of the first dose of the measles vaccine has remained below 95 percent needed to prevent outbreaks. So, this leaves several communities at risk of measles infection [3].

From early 2019, there was a 300% global surge in measles cases compared to the previous year. This increase was remarkably higher in the African region [4] causing many deaths, mostly among infants [3]. Cameroon was one of the countries in this region with high measles activity in 2019 [5]. The measles outbreak touched several areas of the country, including the South West Region (SWR), which has been experiencing armed conflict for over five years [6]. In 2019, this region reported three Health Districts (HDs) that experienced a measles outbreak and a few others that recorded at least one case of measles but did not experience an outbreak.

The SWR has 18 HDs, and each of these HDs is under the leadership of a District Medical Officer (DMO). The HDs are divided into health areas, each led by a chief of the health area. Generally, each health area has at least one health facility, public or private [6]. Also, at least one health facility in each health area is responsible for offering vaccination services to infants [6].

Globally, most settings that experienced an outbreak of measles had low measles vaccination rates [3]. One of the factors that favored the outbreaks was inequitable access to vaccines. A sizable proportion of the world´s infants reside in hard-to-reach communities and areas with civil strife [7]. Countries with high measles vaccination coverage recorded an increased measles incidence in 2019 [5]. Vaccines can lose their potency if not appropriately conserved and within recommended temperatures [8,9]. Given the measles outbreaks in the SWR of Cameroon in 2019, we conducted this study to investigate the factors that favored the epidemic in the affected health districts. The main question was, which factors contributed to the measles outbreak in 2019 in three health districts in the South West of Cameroon? The main objective was to determine the various elements that favored the measles outbreak in the districts. We looked at the role played by the socioeconomic, nutrition, and vaccination status of the people. We also checked vaccine availability, storage conditions, and vaccination coverage´s impact on the outbreak and population movement.

## Methods

**Study area:** Bakassi, Ekondo Titi, and Limbe HDs that experienced an outbreak in 2019 are all located along the Southwest Coast of Cameroon, neighboring Nigeria. These HDs intercommunicate by sea and road to other parts of the region. The other group of HDs is Buea, Muyuka, and Kumba, which reported a measles case in 2019 but did not experience an outbreak. These HDs are neighboring and communicate with the outbreak districts by road.

**Study design:** data were collected from March to August 2020. We conducted a retrospective comparative cross-sectional study in which we reviewed records from January 2015 to December 2019. We assessed population socioeconomic status, vaccine service delivery, vaccination coverage, vaccine storage temperatures, and population movement of the health districts that experienced a measles outbreak and compared their findings to districts that reported a case of measles in 2019 but did not get into an outbreak. The first stage of this study involved reviewing the measles database from the Centre Pasteur Laboratory of Cameroon for 2019 archived in the South West Regional Expanded Program on Immunization. The districts in which measles cases were investigated in 2019 were identified. A district was considered to have experienced a measles outbreak if it reported three or more confirmed measles cases (Immunoglobulin M positive) [10] within 28 consecutive days in 2019. This group of districts was considered the case group. Meanwhile, districts that recorded a confirmed case of measles in one of the months in 2019 without experiencing an outbreak were considered the control.

**Study population:** individuals who resided in any district in SWR in 2019 from where an investigation for measles revealed either a negative or positive immunoglobulin (Ig) M following blood sample analysis done in the Laboratory of Centre Pasteur, Cameroon.

**Sampling method:** the participants or their guardians (for minors) were identified following their measles status in 2019, found in Center Pasteur du Cameroon's South West Regional database obtained from the Delegation of Public Health. We visited these participants in their communities after a phone call or contact by a community health worker. A pretested questionnaire helped us to collect individuals' information. The participants' data from measles outbreak districts were grouped to form the case group, while that from the non-outbreak districts was the control group.

**Selection criteria:** persons with a confirmed measles serology (positive or negative) who resided in any of the districts of interest in 2019 were included in this study. The proof of vaccination was verified through their medical record, and their basic parameters (weight, height, or mid-upper arm circumference) were checked from the records at the time they had the measles. Participants with incomplete medical records were excluded.

**Sample size:** the size of both study groups was determined by convenience. The participants were recruited as they were identified in the database and accessed in the community.

### Study variables

**Measles status:** this was assessed from participants' medical records reported from the Centre Pasteur of Cameroon. The finding was dichotomized as positive or negative. This element indicated that the blood sample for the participant collected in 2019 was positive or negative for measles immunoglobulin (Ig) M.

**Patient outcome:** this was verified through the complication(s) reported by the participant from the onset of the rash and the day of investigation. The result was dichotomized as present or absence or as dead or alive.

**Vaccination against measles:** individual's vaccination against measles was assessed through a vaccination record (card or register)

**Nutritional status:** this was assessed through body mass index (BMI) or mid-upper arm circumference (MUAC). In addition, the receipt of vitamin A supplements in the previous six months was reviewed in the records.

**Socioeconomic status:** this was assessed on a composite scale of monthly income, profession, means of transportation, and ownership of the resident. Since all the study participants were below 18 years old, their socioeconomic status was assessed by considering the status of their parents.

**Movement from a community with a measles outbreak:** reported by subject or guardian as community visited within a month before the onset of symptoms

**Age at diagnosis of measles infection:** reported by study participant and confirmed by patient´s records. Measured on a continuous scale in months and defined as the age at which measles infection was first diagnosed.

**Presence or absence of any chronic disease:** assessed on a dichotomous 'Yes/No' and nominal scales in response to the question, do you (your child) have any chronic illness? If yes, which? The information obtained was confirmed using the patient's record.

### Data collection

**Review of records and selection of cases:** persons with known measles status from the regional database in 2019 were contacted and visited in their respective communities. Their information was entered into a pretested questionnaire with the variables mentioned above. Data collected with the questionnaire included employment status, monthly income bracket, the highest level of formal education attained, and house ownership. Each district's measles vaccination coverage was obtained from the District Vaccine Data Management Tool (DVDMT). Also, each district's records on measles vaccine availability in the previous five years (2015-2019) were checked from their respective stock management tools (SMT). The gap in the available measles vaccine was assessed by comparing the mean available measles-rubella vaccine (formulation used in Cameroon) and the mean of the target population (9 months old) for the study period. The temperatures at which these vaccines were stored were reviewed from the respective archives of the district fridge tag readings.

**Data management and analysis:** data were coded and analyzed with SPSS 20. Before analyses, all continuous data were tested for normality using histogram plots and tests for skewness and kurtosis to justify parametric or non-parametric statistical tests. Continuous data were expressed as means ± standard deviations (SD) and ordinate categories, and non-continuous variables as proportions (%). Univariate analyses were presented as frequencies, means, and standard deviations for continuous variables. The strength of associations between categorical variables was presented as odd ratios with chi-squared tests (X^2^) used to test for statistical significance as well as for differences between proportions. Differences between means of continuous variables were compared between groups with one-way analyses of variance (1-ANOVA). The 1-ANOVA enable us to determine the difference in vaccine exposure to extreme temperatures between the two groups of districts. The vaccination coverage for each antigen was obtained as reported in the DVDMT as the proportion of people vaccinated per antigen expressed as factors of the target population per health area and district. Regression analysis enabled us to determine the contribution of socioeconomic, clinical elements, and travel. All test statistics were two-sided and considered statistically significant at p < 0.05. participants with incomplete data were excluded.

**Ethical considerations:** ethical approval for this study was obtained from the Institutional Review Board of the Faculty of Health Sciences of the University of Buea. Also, administrative authorization was obtained from the Ministry of Public Health, Regional Delegation for the Southwest Region. Comprehensive information about the study was given to the participants before a signed consent was obtained.

## Results

**Sociodemographic characteristics of participants:** a total of 56 participants with known measles status were enrolled in the study: 32 from the outbreak and 24 from non-outbreak districts. Participants were as young as six months and as old as 17 years, with a mean age of 41.1 months (SD - 0.34). There were no statistically significant differences in the sex distribution of the participants from both groups of districts. However, there were more unemployed persons in the HDs that recorded an outbreak than in the districts without an outbreak (9.3% versus 0.0%, p = 0.01) (Table 1). This group of districts without measles outbreaks had a higher education level and monthly income than the outbreak group (Table 1). However, no statistically significant difference was observed between both groups' lodging status (landlord or tenant).

**Table 1:** socioeconomic characteristics of the parents of study participants

	Districts without outbreak		Districts with outbreak		2-sided sig. of difference	Entire study sample	
	n	%	n	%		N	%
**Study participant**	24	42.9	32	57.1		56	100.0
**Occupation**							
Student	2	8.3	1	3.1	0.01*	3	5.3
Unemployed	0	0.0	3	9.3		3	5.3
Housewife	2	8.3	5	15.6		7	12.5
Employed	20	83.3	23	71.9		43	76.8
**Monthly income**							
0-50000frs	10	41.7	27	84.4	0.02*	37	66.1
>50000frs	14	58.3	5	15.6		19	33.9
**Means of transport**							
Pubic	18	75	31	96.9	<0.01*	49	87.5
Private or service	6	25	1	3.1		7	12.5
**Lodging facility**							
Tenant or cohabitate	20	83.3	30	93.8	0.3	50	89.3
Proprietor of home	4	16.7	2	6.3		6	10.7
**Level of education**							
Primary	9	37.5	21	65.6	0.031*	30	53.6
Secondary	7	29.2	7	21.9		14	25
Tertiary	8	33.3	4	12.5		12	21.4
**Marital status**							
Single	8	33.3	10	31.3	1	18	32.1
Married	16	66.7	21	65.6		37	66.1
Widow(ed)	0	0.0	1	3.1		1	1.8

**Clinical and anthropometric assessments of participants:** more participants received vitamin A in the non-outbreak group than in the outbreak group of health districts (92.3% versus 62.5%, p=0.01). There was, however, no statistically significant difference between the MUAC of the participants of both groups (Table 2). We noted equally no statistically significant differences in both groups´ preexisting comorbidity (chronic disease) and the outcomes following measles (Table 2). However, more deaths (0 versus 3) were recorded in the case group.

**Table 2:** clinical and anthropometric assessments of participants

	Measles status				2-sided sig. of diff	Entire study sample		OR (95%CI)
	Districts without outbreak		Districts with outbreak					
	n	%	n	%		N	%	
**Study participants**	24	42.9	32	57.1		56	100.0	
**Nutritional status**								
**Mid-upper arm circumference (MUAC) (cm)**								
Mean±SD	15.2±3.3		15.1±1.2		0.87	16.45 ± 0.8		
<13.5	16	66.7	30	96.7		46	82.1	
≥13.5	8	33.3	02	6.3		10	17.9	
BMI (kg/m^2^)								
Mean±SD	16.98±8.6		18.54±6.6		1	± 0.65		
≥18.5	6	27.3	7	87.5		13	43.3	
<18.5	16	72.7	1	12.5		17	56.7	
**Vitamin A supplementation**								
Received	22	92.3	20	62.5	0.01*	42	75	6.6 (1.31-33.17)
Not received	2	7.7	12	37.5		14	25	
**Measles vaccination**								
Received	19	79.2	31	96.9	0.09	50	89.3	
Not received	5	20.8	1	3.1		6	10.7	
**Chronic disease**								
Yes	1		3		0.463	4		
No	23		29			52		
**Measles status**								
Positive	7	29.2	20	62.5	0.01*	27	48.2	20.1 (7.0-59.0)
Negative	17	70.8	12	37.5		29	51.8	
**Complications after measles infection**								
Yes	1	4.2	3	9.4	0.1	4	7.1	
Death	0	0	3	9.4		3	5.4	
No	23	95.8	29	90.6		32	57.1	

**Vaccine availability as per the need and exposure to extreme temperatures:** vaccines were exposed to extreme temperatures in the outbreak group for a more extended period (Table 3). The mean amount of measles vaccine doses available per district was less than the need in both groups of districts for 2015-2019; for the outbreak group, the vaccine available versus the need was (1705.4 versus 2333.0, p= 0.03), while for the non-outbreak group, it was (3284.4 versus 5206.8, p≤0.01). Compared to the none-outbreak group, the vaccines were exposed to extreme storage temperatures in the HDs with the outbreak (P<0.01) from 2015 to 2019 (Table 3).

**Table 3:** duration of vaccine exposure to extreme temperatures

	Districts without outbreak	Districts with outbreak	2-sided sig. of difference	95% CI
**Meantime vaccines spent in the cold chain above the limit (> 8°C) in hours ± SD**	14.3±38.2	216.5±429.4	<0.01*	123.2- 281.1
**Mean time below limit (-0.5°C) in hours ± SD**	1.9±14.5	26.2±95.4		6.5- 41.9
**Minimum Temperature (°C) of cold chain**	1.6±1.9	0.1±2.2		-2.1 - -0.9
**Maximum temperature (°C) of cold chain**	11.7±5.9	46.4±244.1	0.1	

**Figure 1:**
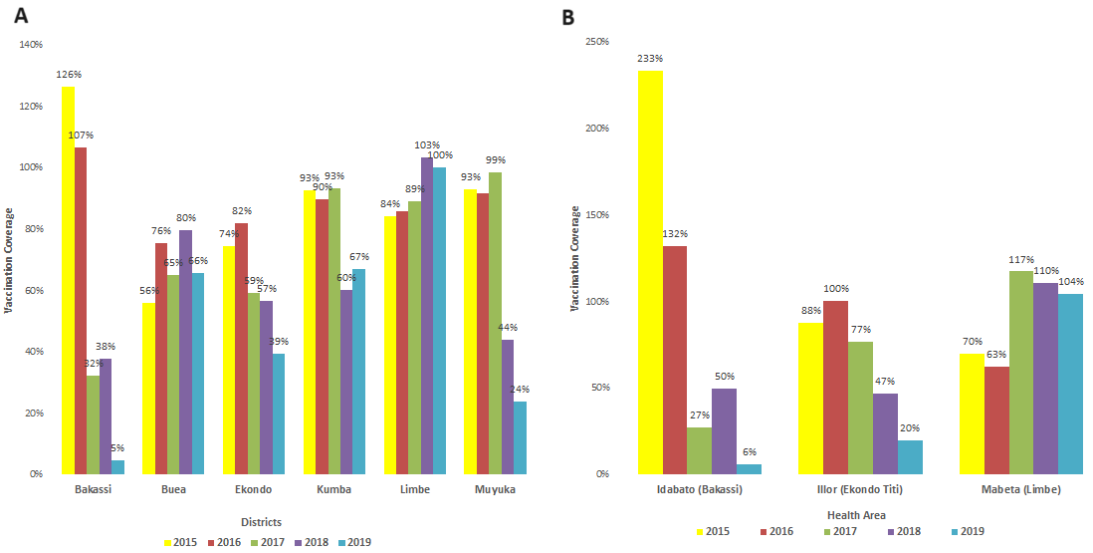
A,B) evolution in measles vaccination coverage from 2015 to 2019

**Vaccination coverages:** there was a downward trend in the vaccination coverage in the health districts in the outbreak group, except for the Limbe Health District. However, the Mabeta health area, the site of the epidemic in Limbe HD, also presented a downward trend in measles vaccination coverage (Figure 1).

**Travel history from sites in the measles outbreak:** Table 4 shows the traveling tendency of people in both groups of districts within the previous 30 days before the onset of the disease. The first travel case was a participant who returned from Nigeria in March 2019 to Bakassi HD and developed a rash a few days later. This infant tested positive for measles.

**Table 4:** population movement from sites in the measles outbreak

	Measles status				2-sided sig. of difference	Entire study sample		OR (95%CI)
	Districts without outbreak		Districts with outbreak					
	n	%	n	%		N	%	
**N**	24	42.9	32	57.1		56	100.0	
**Travel history**								
Yes	34		18		<0.01*	16	28.6	2 (1.1-11.20)
No travel	23	95.8	15	46.9		40	71.4	
**Travel from**								
Nigeria	0	0	14	40.6	<0.01*	14	25	38.1 (2.4-68.0)
Ekondo Titi	0	0	1	59.4		1	1.8	2.7 (2.4-68.0)
Limbe	1	4.2	1	6.3		1	1.8	1.6 (2.4-68.0)

**Evolution of the security profile and service delivery:** the entire SWR had been experiencing insecurity since 2016, following the onset of a sociopolitical crisis that finally degenerated into full-blown armed violence. Bakassi, Ekondo Titi HD, and the Mabeta health area (in Limbe HD) that experienced the outbreak were highly insecure (though Limbe was considered moderate insecurity). Meanwhile, in the non-outbreak group, Buea was moderately insecure, while Kumba and Muyuka were highly insecure. None of the three district health services in the outbreak group were fully functional. They all operated remotely, while in the non-outbreak group, 2 (67%) were fully functional, while one functioned remotely. In addition, unlike in 2015, when all the vaccinating health facilities were fully operational in both groups of districts, in 2019, it was observed in the outbreak group that just 32(63 %) were fully functional against 51(76%) in the non-outbreak group due to arms conflict. There was also a drop in the proportion of staff offering immunization services in all these districts. Both groups' mass supplementary immunization activities dropped from 3 in 2015 to 1 (33%) in 2019.

## Discussion

The SWR of Cameroon has been in armed conflict since late 2016. This issue resulted in mass population displacement, distorted the health systems, and interfered with essential health services like vaccination [6,11]. Like other parts of the country, this region experienced increased laboratory-diagnosed measles cases in 2019 [12]. The rise in measles cases that touched several districts of this region was an added challenge, provoking a measles outbreak in three districts with an already weakened health system following the armed conflict [6,13]. This fragile health system was later added to the coronavirus disease (COVID)-19 burden in 2020 [14]. The challenge in this region prompted us to assess the specific factors associated with the measles outbreak in the three affected districts.

It was observed that the population in the outbreak group had a lower socioeconomic status (Table 1). Furthermore, both districts had suboptimal vaccine availability compared to the needs. Also, there was a downward trend in measles vaccination coverage in all the affected communities from 2015 to 2019 (Figure 1). The vaccines were exposed to extreme temperatures in the districts with the outbreak for extended periods. People in the outbreak group were more likely to have traveled from an area in measles outbreak OR 2(0.03-11.20), like Nigeria, before the onset of measles (Table 4).

Most of the participants in the outbreak districts had a lower socioeconomic status and just primary education than those in the none-outbreak group who had higher socioeconomic status and received a secondary or tertiary education (p=0.01). These factors could favor measles infection and outbreaks since the guardians may tend to incline more toward traditional beliefs on the cause and cure of measles and pay less attention to vaccination and care [15]. Vaccination coverage dropped in all the districts. This issue could be partly linked to the drop in the proportion of health facilities offering vaccination services with the evolution of the armed conflict. Vaccines were also less available than needed in both districts, partly due to challenges in shipment and closure of health facilities following the insecurity [16]. This likely made it difficult for all the available infants to get vaccinated. This gap in vaccine availability could further contribute to the fall in vaccination coverage observed over the years. A drop or low measle vaccination coverage favored the measles outbreak [17,18]. Annual mass vaccination campaigns in the region that would have given more opportunities for infants to receive the vaccines and avoid missed doses became very uncommon. Vaccination campaigns were suspended partly due to violence [19], looting of health facilities, killing of health staff, and population movement [6,20].

Vaccines were more exposed to extreme temperatures in the outbreak districts. This situation could have caused the available vaccines to lose potency [15]. So, such vaccines might not have provided the population with the expected protection [9]. Of particular interest is the measles-rubella vaccine used in this setting, which is more thermolabile and may be destroyed after 14 days if exposed continually to a temperature of about 37°C [21]. This time is shortened if the vaccine is exposed to a higher temperature. Although Cameroon has begun reinforcing its vaccine cold chain by providing health facilities with prequalified cold chain equipment, it is urgent to hasten the deployment in this war-tone region to help prevent similar outbreaks. Using local actors accepted in this setting may help improve access to install equipment and carry out vaccination services [22]. In addition, implementing supplementary immunizations and periodic intensification of routine immunization activities during periods of relative calm will help reach more missed infants and improve vaccine uptake [19,23]. Also, traveling from an area during a measles outbreak was more common in the outbreak group. A participant in Bakassi traveled from Nigeria in March 2019, just before the diagnosis of measles. Measles outbreaks were reported in February 2019, touching at least five states of Nigeria, including Cross River State, neighboring the SWR of Cameroon [24,25]. This movement was likely a factor that favored the propagation of measles in this district [26].

To the best of our knowledge, this is the first research that elucidates the factors associated with the measles outbreak in this region of Cameroon hit by armed conflict for over six years now. Despite the merit of this work, the primary data for this research was collected in the context of insecurity. This situation might have introduced some bias to the report. Also, the denominators used in all calculations were based on national estimates, which may not necessarily reflect the actual situation in the field, considering the current population movements due to the crisis. The sampling method limited our assessments to the participants who could be reached by phone or by community health workers. In addition, the evaluation of the vaccine storage temperature was limited to just the district cold chain. This evaluation does not give further information on the vaccine storage condition in health facilities. Further research may help assess the vaccine storage at health facilities and the immune response of those who have received measles vaccines from these communities.

## Conclusion

Our findings identified vital factors contributing to the measles outbreak in three districts of the SWR of Cameroon in 2019. These factors included low socioeconomic status, sub-optimal vaccine availability, storage of measles vaccines in extreme temperatures, low vaccination coverage, and population movement from sites in the measles outbreak. Adopting strategies to improve the socioeconomic status, vaccine uptake, reinforcing vaccine storage conditions, and frontier disease surveillance in this context of insecurity will prevent similar epidemics in these districts of Cameroon.

### What is known about this topic

- *Measles is a highly infectious viral disease that affects susceptible individuals of all ages;*
- *Due to suboptimal vaccination coverage, this disease is a leading cause of death among young children globally;*
- *In 2019, there was a 300% global surge in measles cases compared to the previous year, and this increase was remarkably higher in the African region*.

### What this study adds

- *Measles affected districts in insecurity in the SWR of Cameroon in 2019;*
- *The health districts affected by measles had low vaccine availability and coverage, and the vaccines were stored in extreme temperatures that could denature MR vaccines;*
- *Reinforcing vaccine storage conditions, adopting strategies to improve vaccine uptake, and disease surveillance in this region hit by armed conflict will help prevent similar outbreaks*.
